# Sleep, Activity, and Well-Being In Persons With Dementia: Findings From the Healthy Patterns Trial

**DOI:** 10.1093/geroni/igab046.2090

**Published:** 2021-12-17

**Authors:** Nancy Hodgson, Darina Petrovsky

## Abstract

Irregular sleep-wake patterns are common in persons living with dementia (PLWD), pose a great burden to caregivers, and are the principal causes of distress and institutionalization of PLWD. A growing body of research supports the importance of activity-based interventions to reduce the frequency and intensity of sleep wake disruption, reduce neuropsychiatric symptoms, and improve quality of life. To date, there are no studies linking sleep disruption and well-being with the nature and timing of activity. This session focuses on lessons learned from the Healthy Patterns Study - a randomized trial of a home-based activity intervention in 200 dyads of PLWD and their caregivers (NCT03682185). Session 1 focuses on the main findings from the clinical trial. Session 2 focuses on the cultural adaptation of the timed activity protocol to improve quality of life (QOL), improve sleep and reduce neuropsychiatric symptoms in older Latinos Session 3 describes the community outreach efforts used over a one-year period to recruit a diverse sample of PLWD and their caregivers for the Healthy Patterns trial. Session 4 examine the relationship between caregiver mastery and neuropsychiatric symptoms in PLWD. Together these findings highlight the complex role of sleep and wake activity in promoting well-being in persons with dementia.

